# RulNet: A Web-Oriented Platform for Regulatory Network Inference, Application to Wheat –Omics Data

**DOI:** 10.1371/journal.pone.0127127

**Published:** 2015-05-19

**Authors:** Jonathan Vincent, Pierre Martre, Benjamin Gouriou, Catherine Ravel, Zhanwu Dai, Jean-Marc Petit, Marie Pailloux

**Affiliations:** 1 Blaise Pascal University, UMR6158 CNRS LIMOS Laboratoire d'Informatique, de Modélisation et d'Optimisation des Systèmes, Aubière, F-63 173, France; 2 INRA, UMR1095 Genetics, Diversity and Ecophysiology of Cereals, Clermont-Ferrand, F-63 039, France; 3 Blaise Pascal University, UMR1095 Genetics, Diversity and Ecophysiology of Cereals, Aubière, F-63 177, France; 4 INSA Lyon, UMR5205 CNRS LIRIS Laboratoire d’Informatique en Images et Systèmes d’Information, Villeurbanne, F-69 621, France; Aberystwyth University, UNITED KINGDOM

## Abstract

With the increasing amount of –omics data available, a particular effort has to be made to provide suitable analysis tools. A major challenge is that of unraveling the molecular regulatory networks from massive and heterogeneous datasets. Here we describe RulNet, a web-oriented platform dedicated to the inference and analysis of regulatory networks from qualitative and quantitative –omics data by means of rule discovery. Queries for rule discovery can be written in an extended form of the RQL query language, which has a syntax similar to SQL. RulNet also offers users interactive features that progressively adjust and refine the inferred networks. In this paper, we present a functional characterization of RulNet and compare inferred networks with correlation-based approaches. The performance of RulNet has been evaluated using the three benchmark datasets used for the transcriptional network inference challenge DREAM5. Overall, RulNet performed as well as the best methods that participated in this challenge and it was shown to behave more consistently when compared across the three datasets. Finally, we assessed the suitability of RulNet to analyze experimental –omics data and to infer regulatory networks involved in the response to nitrogen and sulfur supply in wheat (*Triticum aestivum* L.) grains. The results highlight putative actors governing the response to nitrogen and sulfur supply in wheat grains. We evaluate the main characteristics and features of RulNet as an all-in-one solution for RN inference, visualization and editing. Using simple yet powerful RulNet queries allowed RNs involved in the adaptation of wheat grain to N and S supply to be discovered. We demonstrate the effectiveness and suitability of RulNet as a platform for the analysis of RNs involving different types of –omics data. The results are promising since they are consistent with what was previously established by the scientific community.

## Introduction

Regulation of gene expression is defined as the spatiotemporal control of the amount of gene products. It governs cell differentiation and the adaptation of living organisms to their environment. Out of the diverse levels of regulation, transcriptional control is considered crucial and is often conserved in both eukaryotes and prokaryotes. Regulatory sequences upstream of genes together with proteins able to recognize and bind specifically to these sequences, called transcription factors (TFs), were substantiated in the late 1960’s [1]. TFs are themselves encoded by genes and the complex interactions between genes and TFs put together are called Gene Regulatory Networks (GRNs). GRNs have been inferred efficiently in the past decade, leading to significant breakthroughs [2–5]. However, GRNs are not always sufficient to explain the complex physiology of a living system. A wider range of regulatory relationships between genes, their products and their interactions with the various metabolites and signaling molecules present in a cell are generally considered necessary to explain this complexity. These are called Regulatory Networks (RNs).

Inference of RNs is therefore a promising approach to elucidating the complexity of molecular interactions. Existing RN inference approaches generally use machine learning methods and can be categorized according to the strategy employed [6] as supervised [7] or unsupervised [8] i.e. whether learning samples are necessary or not, and whether all (global) or only a portion (query-driven) of the hypothetic relationships are inferred. Query driven approaches allow prioritizing predictions likely to be valuable in the context of a research. These approaches can be further divided according to the data mining or statistical methods they rely on to infer interactions. The most widely used approaches are Boolean networks [9, 10], Bayesian networks [11], Petri nets [12, 13] and association rule discovery[14–16].

Several tools based on association rule discovery have been developed. For instance, GenMiner [16] allows the simultaneous analysis of biological datasets such as gene expression or annotation, but quantitative data are preprocessed and discretized in the workflow. Georgi *et al*. [15] has presented an approach for association rule discovery that uses quantitative data and allows users to specify parameters to discover rules matching their personal interest. These systems do not, however, provide an integrated framework for the visualization and editing of inferred networks which would be of more use to most research biologists. A small number of web-oriented platforms are available to infer RNs and GENIES [17] for instance provides a supervised method to construct networks using partially known network information. It allows loading heterogeneous data but lacks visualization features for the resulting networks. Predictive Networks [18] is an all-in-one web-oriented platform with visualization and analysis tools. Its workflow consists of a data- and text-mining pipeline combined with a seeded Bayesian network inference method.

In this paper we present RulNet as a novel integrative, query driven approach using a relational data mining method extended from Agier *et al*. [14]. It is implemented as a web-oriented platform dedicated to the inference of RNs suitable for qualitative and quantitative—omics data. It provides users with options to specify entities (genes, metabolites,…) of interest to discover rules involving these entities. A special query language has been integrated into RulNet which allows users to perform custom queries. Each type of rule has its own biological interpretation, which provides flexibility in the type of networks that can be inferred. RulNet can therefore be used to infer very distinct networks e.g. between traits and genetic markers in association studies. This all-in-one platform allows experimental data to be uploaded or data already in a database management system (DBMS) can be interrogated. The RulNet platform has several functionalities such as validation or invalidation of interactions, network generation from one or two lists of components and parameterization of the number of intermediate components. The ultimate aim of RulNet is to provide biological researchers with more control over their search for interactions during the network inference process.

In order to assess the effectiveness and suitability of RulNet to infer RNs, we first benchmarked it using the standard datasets used for the transcriptional network inference challenge DREAM5 [19]. We then used RulNet to analyze the changes in gene expression, grain storage protein (GSP) composition, free amino acids and key metabolites concentrations in response to nitrogen (N) and sulfur (S) supply for developing wheat (*Triticum aestivum* L.) grain. Wheat GSPs accumulate during the effective filling phase of grain development and account for 60% to 80% of the total protein content of mature grains [20]. Increased N supply via the use of fertilizers increases grain yield and protein concentration [21], but also modifies the balance between GSP fractions, i.e. gliadins and glutenins, the most important single trait determining wheat end-use value [22, 23]. Concurrently, S is essential for an efficient use of N and to maintain balanced GSP composition [24, 25]. The RN inferred highlights genes responsive to N and S supply as well as their links with various GSP fractions and metabolites.

RulNet is publicly available at http://rulnet.isima.fr. Users can also download and run the application on their own servers or computers.

## Methods

### Multipredicate rules and RQL language

The notion of rules or implications is widely used in the database, data mining and artificial intelligence communities. A rule reflects an observation on the data. Examples of rules are functional dependencies [26], implications [27] or association rules [28]. A rule is written *X*→*Y* and is read *X* implies *Y*, where *X* and *Y* are two sets of attributes (i.e. entities). A rule expresses a new relation when a property (called a predicate) is observed on *X* (the left hand side or antecedent) then a property is observed on *Y* (the right hand side or consequent) in the data. The two predicates can be of the same type but not necessarily.

The principle of a rule-based learning approach is to indicate which type of rules (semantics) to discover then to apply algorithms to infer all the rules satisfying this semantics in the data. The biologist then needs to interpret the set of rules inferred and determine if they have a biological meaning. The intrinsic variability (from noise and error) of biological data means that learning approximate rules, i.e. rules that are not always satisfied in the data is also of interest.

A generic SQL-like language called RQL (Rule Query Language) has been recently developed to specify rule semantics in a simple way [29]. RQL allows association rules or functional dependencies on a relation to be defined but can also capture rules between attributes from different tables of a database, or conditional rules such as conditional functional dependencies [30, 31]. As originally defined, RQL is however limited to rules with a single predicate (i.e. the same condition on all the attributes of the left hand side implies the same condition on all the attributes on the right hand side). As an example, for exact association rules from a binary relation, the predicate asserts that the attribute has to be equal to 1 (*AB*→*C* if for all samples, when *A* = 1 and *B* = 1, then *C* = 1). For functional dependencies, the predicate asserts the equality between two values (*AB*→*C* if for all couples of samples with a same value for *A* and a same value for *B*, then *C* has a same value).

This restriction to a single predicate does not allow all the behaviors captured by the data to be inferred, in particular from biological data. Moreover, if users want to work with different types of attributes (quantitative, categorical or binary) a single predicate may not be used for all attributes. Therefore, we have extended RQL so that multipredicate rules (MP-rules) can be defined and generated. Each predicate can have a different meaning corresponding to a specific objective for the application domain and the type of attributes. The advantage of MP-rules, is that we can capture different perspectives from the data. The advantage is also that we can address quantitative, categorical or binary attributes in the same inference process.

**Fig 1:**
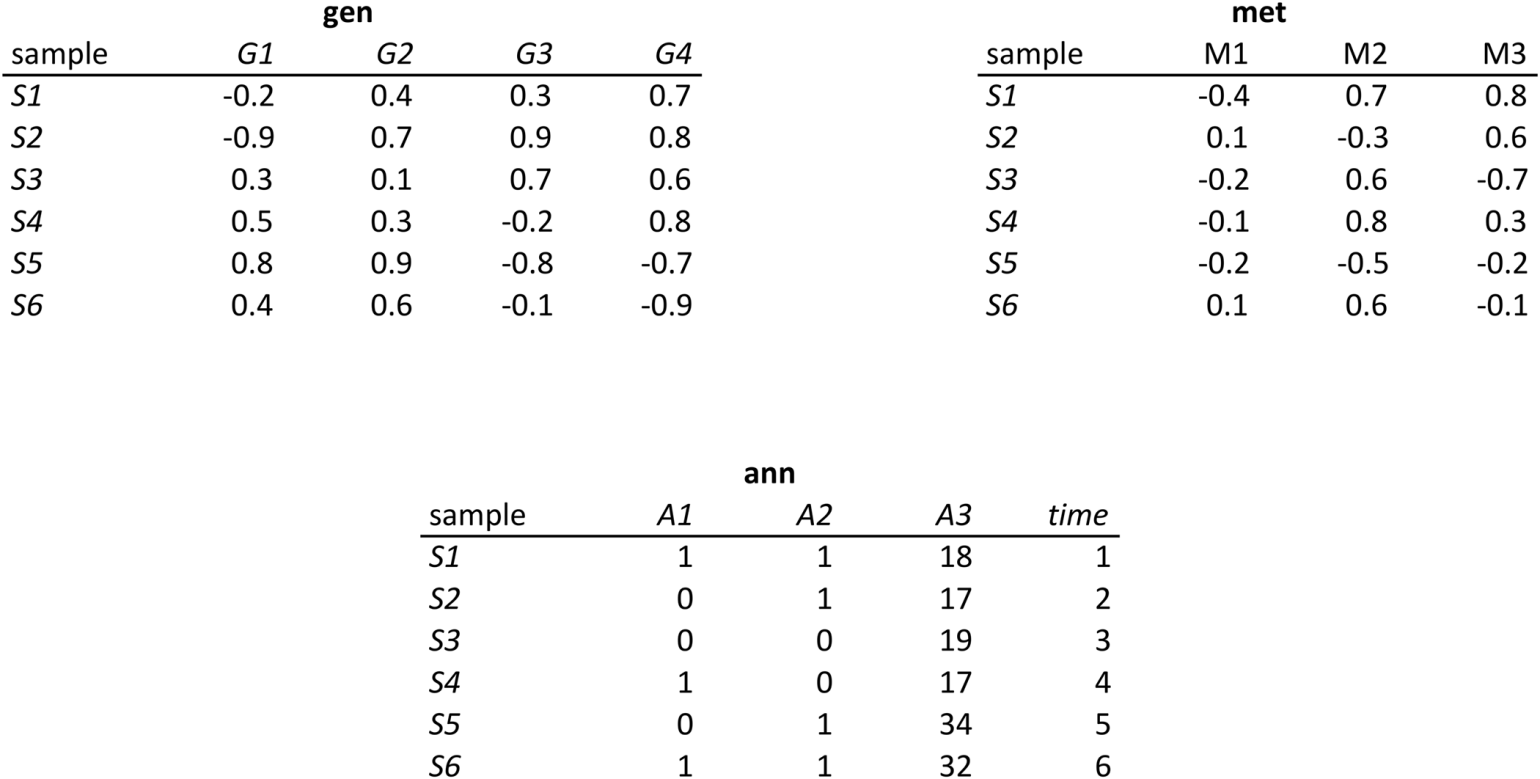
Example of a database composed of three tables. Table gen (gene expression), met (metabolites assay) and ann (sample annotation), containing heterogeneous data for six samples. These tables contain quantitative attributes (*G*1, *G*2, *G*3, *G*4, *M*1, *M*2, *M*3, *A*3), binary attributes (*A*1, *A*2) and categorical attributes (time).

To illustrate the use of MP-rules, let us consider a dataset composed of three tables (Fig 1). In this dataset, each time *A*1 = 1 in table ann then *M*2 > 0.5 in table met. This observation can be represented by the MP-rule *A*1_1_→*M*2, which means that *A*1 = 1 guarantees *M*2 > 0.5 (i.e. *A*1 = 1 is a sufficient condition to observe *M*2 > 0.5). Another exact MP-rule in this dataset is *G*3→*A*1_0_ (i.e. if *G*3 > 0.5 then *A*1 = 0). Here *A*1 = 0 is a necessary condition to observed *G*3 > 0.5.

In RulNet, a query for MP-rules has the following template:

FINDRULES

 SCOPE t1 IN (dataset1), t2 IN (dataset2), …

 WHERE condition(t1, t2, …)

 HAVING label1: predicate1(t1, t2, …) OVER attributes1

 AND label2: predicate2(t1, t2, …) OVER attributes2

 AND …;

where the "SCOPE" line defines the tuples (rows) of the datasets on which the "HAVING" clause is tested. Datasets can be data files, database tables or SQL queries. The "HAVING" and "AND" lines define the predicates and the associated lists of attributes, respectively. The "WHERE" line is optional, it allows setting conditions on the tuples. For instance, in the dataset of Fig 1, the clause WHERE *t*2.time = *t*1.time + 1 means that all the couples of consecutive samples (tuples) will be tested. The theoretical basis for rules expressed in RQL and implemented in RulNet has been presented by Chardin *et al*. [29] but these theoretical aspects are beyond the scope of this paper.

### Rule generation and interest measures

In RulNet, users have the possibility to generate both exact and approximate 1–1 MP-rules (e.g. *G*1→*G*2). Users can also choose to infer a subset of n-1 MP-rules (e.g *G*1*G*2→*G*3). In this case, considering the very large number of possible rules, only the exact MP-rules with the smaller left hand sides (in other words, the direct interactions) and the approximate MP-rules with the larger left hand sides are generated. This corresponds respectively to the canonical and the Gottlob and Libkin covers [32] generalized to MP-rules. The first step of the process consists of generating an intermediate set from which the rules will be deduced. This set is a generalization to MP-rules of a result given for functional dependencies [33] and for well-formed semantics [14]. In RulNet, the set generation can be accomplished with only a single SQL query and thus benefit from the optimization features available in relational DBMS [29, 34]. The second step for the generation of n-1 MP-rules, consists of computing the two sets of exact and approximate MP-rules [35]. This is the most expensive step in terms of computation time. RulNet uses the algorithm and the code proposed by Murakami and Uno, which is recognized as the best solution for such problems [36]. Once generated, rules can be objectively evaluated by calculating quality measures. Support, confidence, lift and leverage are four commonly used measures of significance and interestingness for association rules [37, 38]. In RulNet, these metrics were adapted for MP-rules and are defined as follows.

The support is a measure of significance (importance) of a rule and is given by:

$$Support(X\to Y)\;=\;\frac{\text{count of }(X\cup Y)}{N}$$ (1)

where N is the number of possible tuples in the dataset verifying the specified condition if it exists (the “WHERE” clause) if only one tuple variable (t1) is defined. If two or more tuple variables are specified, N is equal to the number of possible combinations of tuples in each dataset verifying the specified condition if it exists. The support is often used to reduce the search space (i.e. to retain only the rules with a support higher than a user defined threshold value). The confidence is a measure of the strength of a rule. It is defined as the probability of seeing the rule's consequent under the condition that the antecedent is satisfied. The confidence is directed and gives different values for the rules *X*→*Y* and *Y*→*X*. The confidence is given by:

$$Confidence(X\to Y)\;=\;\frac{\text{count of }(X\cup Y)}{\text{count of }X}$$ (2)

The lift [39] and the leverage [40] compare the observed support of the rule and the expected support if X and Y are statistically independent. The lift compares how many times X and Y occur together with the number of times they would occur together it they were statistically independent:

$$Lift(X\to Y)\;=\;\frac{\text{count of }(X\cup Y)\;\times \;N}{\text{count of }X\;\times \text{ count of }Y}$$ (3)

The leverage measures the difference of X and Y appearing together in the dataset and what would be expected if X and Y were statistically dependent:

$$\begin{array}{l} Leverage\left(X\to Y\right)=\frac{\text{count of }\left(X\cup Y\right)}{N}\\ \;-\frac{\text{count of }X}{N}\times \frac{\text{count of }Y}{N} \end{array}$$ (4)

A lift equal to 1 and a leverage equal to 0 means that X and Y are statistically independent.

### Workflow

**Fig 2:**
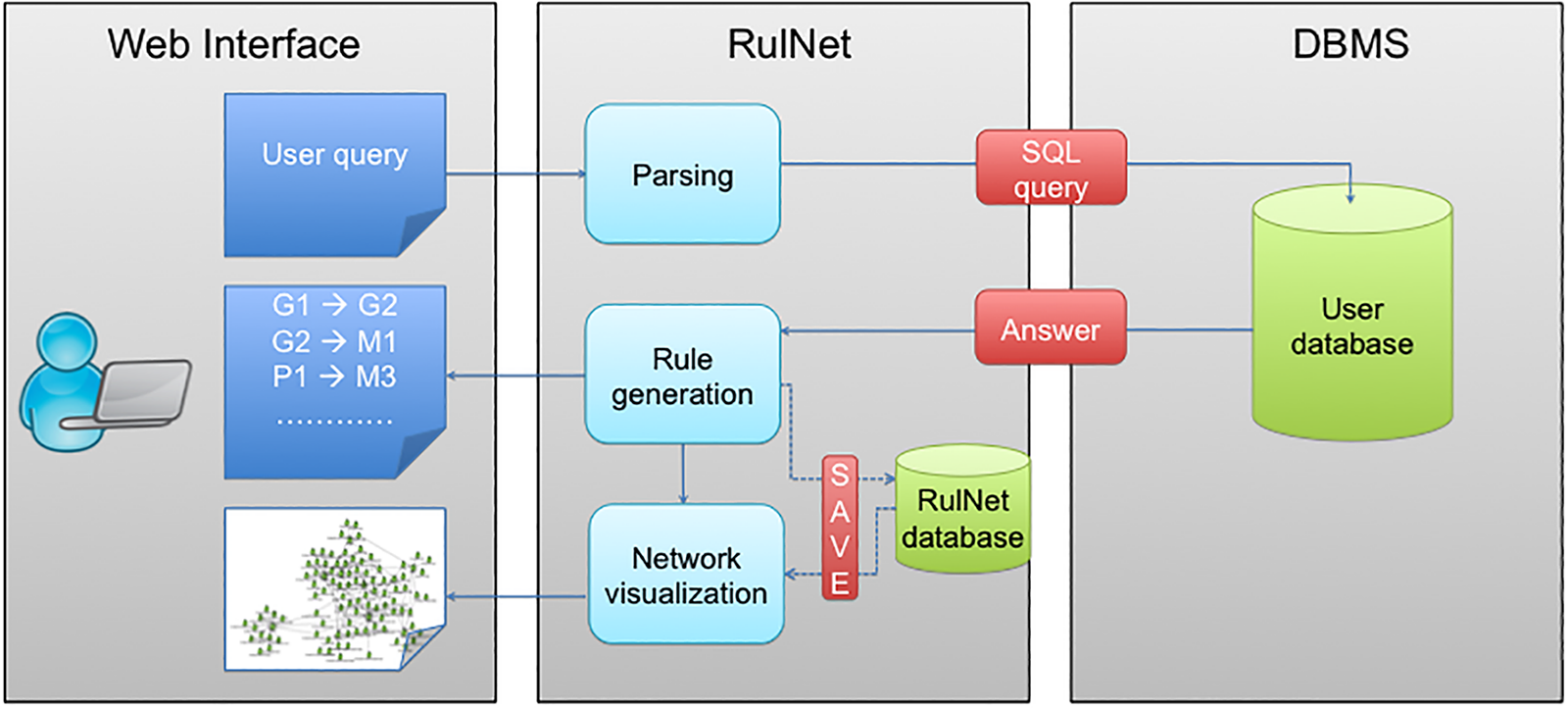
Scheme of the workflow of the RulNet platform and leading to the discovery and visualization of rules. The overall workflow from user’s point of view consists of three steps that are data upload, query design and visualization and edition of inferred networks. These steps can be saved and reloaded afterwards when using a registered account.

The steps leading to the establishment of a rule network are described in Fig 2. Users can upload their data via the web interface as tab-delimited text files that contain samples (rows) and attributes (columns). Each file is then parsed and turned into a table of a database. RQL is coupled with the standard SQL language; therefore, users can skip the file upload step by connecting directly to a database by specifying the corresponding parameters. The advantage is that the data can be queried directly from where they are stored without any complex preprocessing task. The second benefit is that it takes advantage of DBMS technologies for the optimization of query performance and hence computation time.

The second step consists of specifying queries that define the semantics of the rules to generate. A syntactic parsing step is implemented to verify that queries respect the defined Backus-Naur form standards [41] (S1 File) and a query builder is proposed to facilitate the understanding of the language. Two commonly used queries are predefined in the query builder. The first one (called Q1) is similar to association rules and discovers rules between entities if they show similar high or low values in the same samples. The second one (called Q2) generates rules between attributes showing similar profiles in the data. For each query, users can specify the parameters and the thresholds to use for the quality measures.

Networks of generated rules can be visualized with different layouts and the platform offers different features to manipulate these networks. For instance, rules obtained using different queries can be visualized on the same network. Registered users are given the possibility to save and reload the rules and networks inferred at different steps of the analysis. Generated rules can be exported as a Cytoscape 3.0 compliant tab-delimited file for further analysis or to enhance the display of the networks in Cytoscape, a popular software for visualization and analysis of biological networks [42, 43].

### Characteristics and features

RulNet is available as a web-oriented platform (http://rulnet.isima.fr) installed on a Transtec 2300L Data Storage Server running Intel Xeon E5506 quad-core 2.13 GHz processors with 4 Mb of L2 cache and 8 Gb of RAM. The form parts are implemented using the framework Richfaces 3.3.2 from JBOSS that simplifies the using of AJAX with JSF 1.2 technology. The display layer is a Java applet, built with the JDK6 version. Users can also download the application to install it on their own server or local machine.

### Computational performance

**Fig 3:**
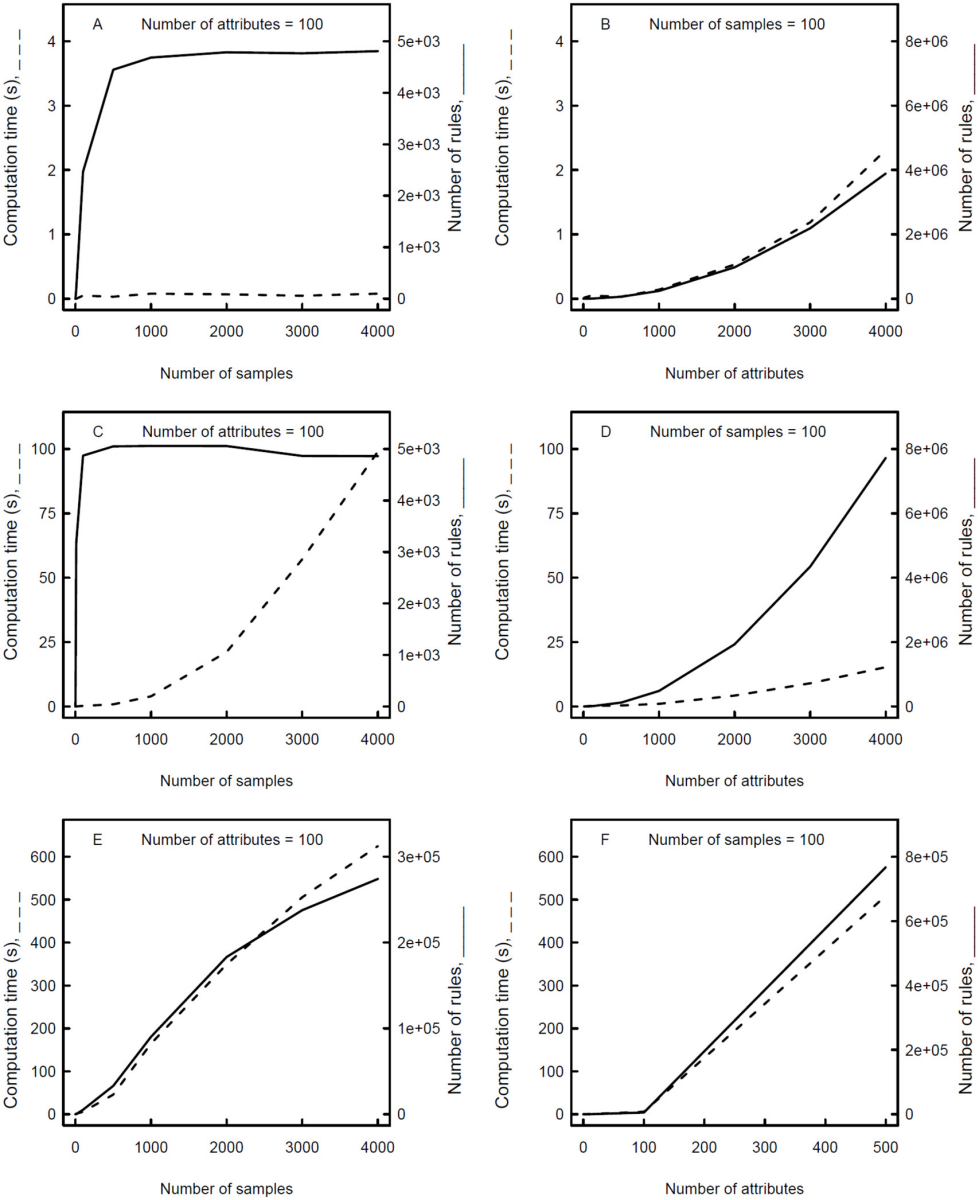
Computing time and number of rules of networks inferred with the RulNet platform. Computing time (dashed lines) and number of rules (solid lines) for query Q1 (A, B) and Q2 (C, D) for 1–1 rules and for query Q1 for n-1 rules with n ≤ 3 (E, F) applied to a randomized dataset with 10 to 4,000 samples (A, C, E) and attributes (B, D, F).

In order to assess the computational performance of RulNet, the two predefined queries were run on the platform using datasets consisting of randomized numerical data. Computing times and the total number of rules discovered (exact and approximate rules) were recorded for datasets of 10 to 4,000 samples (with 100 attributes) and 10 to 4,000 attributes (with 100 samples; Fig 3). The number of rules given is only indicative as it could vary depending on the dataset used.

For Q1, computing time is low (< 3 s) and does not increase significantly with the number of samples (Fig 3A). For Q2, computing time is substantially higher than for Q1 (Fig 3C), because two tuple variables are defined in Q2, which increases with the number of samples. The number of rules discovered increases with the number of samples and is maximum for 500 and 100 samples for Q1 and Q2, respectively. For Q1 and Q2, both computation time and the number of rules increase with the number of attributes (Fig 3B–3D). The number of rules scales with the number of attributes, with a scaling exponent (γ) equal to two.

With query Q1 the number of n-1 MP-rules (n ≤ 3) and associated computing time increase steadily with the number of samples (Fig 3E). Computing time increases steeply for 1,000 attributes and exceeds the session timeout of the web server and results are reported for up to 500 attributes only (Fig 3F). As for 1–1 rules, the number of rules scales with the number of attributes, but the scaling exponent is 50% higher than for 1–1 rules.

## Results

To illustrate the results that can be obtained with RulNet, the platform was used to mine the results of an experiment carried out to better understand wheat grain development adaptive responses to N and S supply (Z. Dai *et al*., in prep.).

Vernalized plants of the winter bread wheat cultivar Récital were grown in a growth chamber from growth stage 13 [44] to ripeness maturity in a modified Hoagland’s nutrient solution [45] with different rates of N and S supply, either 3 mM N and 2 mM S (control treatment), 3 mM N and 0.02 mM S (low S treatment), or 15 mM N and 0.02 mM S (low S and supra-optimal N treatment). Three additional treatments were applied to each of the three main treatments, complementing the nutrient solution with N and/or S from 25 days after flowering (i.e. midway through the effective grain filling phase) to grain ripeness maturity. Grains were sampled every 3 to 5 days between 10 (i.e. two thirds through the lag phase of grain development) and 34 (i.e. physiological maturity) days after flowering for each of the three main treatments. Grains from the six treatments were also sampled at 0, 6, 9, 12, 18, 24, 48 and 72 h after the shift in N and S supply.

Three independent replicates were used and the following measurements were made for each sampling date. Gene expression was monitored using a custom 40k Nimblegen wheat microarray [46]. Transcriptomic data are available from ArrayExpress repository under the experiment names “N and S regulation of wheat storage protein accumulation” (accessions E-MTAB-1782) and “short-term effect of N and S supply shifts on gene expression in wheat grain” (accessions E-MTAB-1920). The main grain storage protein (GSP) fractions contributing to wheat bread making quality are glutenins and gliadins. High (HMW-GS) and low (LMW-GS) molecular weight-glutenin subunits and ω1,2-, ω5-, γ- and α/β-gliadin proteins were separated and quantified by RP-HPLC [47]. The concentration of free amino acids was also assayed by RP-HPLC [48]. Metabolites, including organic acids (malate and citrate), soluble sugars (glucose, fructose, and sucrose), oxidized and reduced glutathione and starch were assayed as described by Gibon *et al*. [49].

In the context of this experiment, we defined two distinct objectives. Firstly we wished to compare the topology of networks obtained using RulNet with those obtained using two widely used RN inference methods; these being a straightforward Pearson’s correlation- based approach (hereafter Pearson) and the Weighted Gene Co-Expression Network Analysis (WGCNA) [50, 51]. Secondly, we wished to illustrate the use of RulNet as a biology-driven clustering method.

### Topological comparison of correlation-based and RulNet regulatory networks

Using the Significant Analysis of Microarrays (SAM) dimension reduction technique [52] we identified a total of 984 transcripts associated with N and/or S supply out of the 40,642 transcripts spots on the microarray. Among these transcripts, 96 were associated with N supply, 237 with S supply, and 640 with both N and S supply. The expression data for these 984 transcripts were used to infer undirected RNs.

For the three RN inference methods performed here, the only preprocessing steps were that data were scaled and centered. Threshold parameters for the three methods were set using the scale-free criterion (S1 Fig). A common property of large non-random networks is that the distribution of their local connectivity is free of scale, following P(k) ~ k-γ, where P(k) is the probability that an attribute in the network interacts with k other attributes [53]. Pearson RNs were generated using a distance matrix computed using Pearson’s correlations with a cutoff threshold of 0.8. In RulNet, we used the Q1 query from the query builder with Confidence and Support thresholds of 0.90 and 0.10, respectively. WGCNA RNs were inferred using the R package WGCNA version 1.34 [51] with power adjacency set to 9.

**Fig 4:**
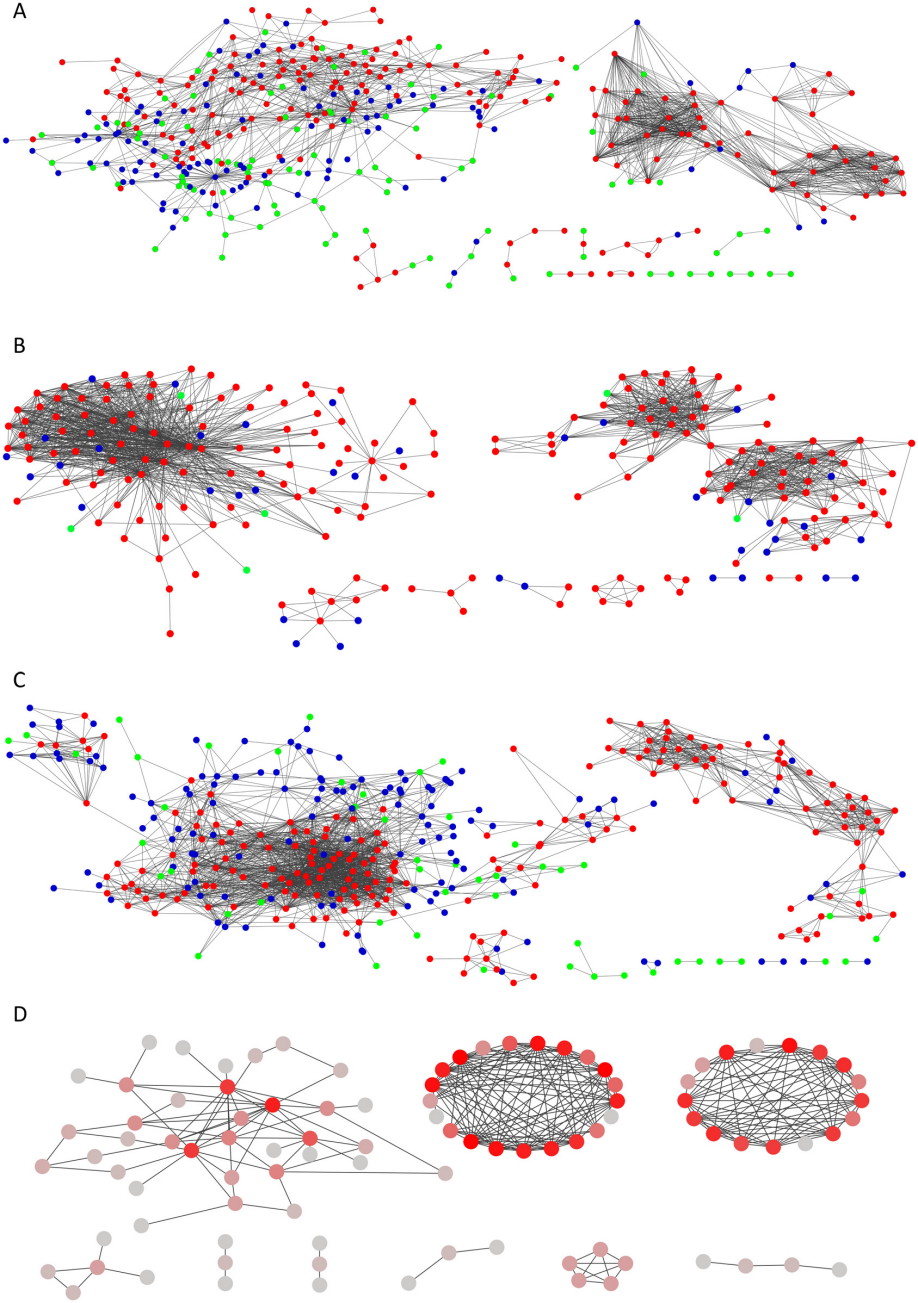
Undirected scale-free regulatory networks inferred with the RulNet platform. (A) and the GCNA (B) and Pearson (C) methods and consensus network obtained using the intersection algorithm of Cytoscape (D). In A, B, and C node color indicate the conservation between the three methods: green, nodes specific to the network; blue, nodes common with one other network; and red nodes found with all three methods. In D, node color is dependent on node degree, from gray for less connected nodes to red for nodes showing the highest connectivity. To enhance the clarity of the figure, disconnected nodes and connected components involving two nodes or less are not shown.

The three methods show common features as well as topological differences (Fig 4, Table 1). For the three methods, the scaling exponent of the power law distribution is lower than 2 (Table 1), indicating a high importance of the hubs in the networks [19]. The number of nodes is similar for Pearson and RulNet but is 38% lower for WGCNA, while the number of edges is similar for RulNet and WGCNA but is 60% higher for Pearson. The network connectivity assessed by the average number of neighbors (i.e. the edge-to-node ratio) is preserved between Pearson and WGCNA, but is lower for RulNet (Table 1). It results in a slightly higher number of connected components for the correlation-based methods than for RulNet. Both correlation-based methods have a higher tendency to create “community structures” than RulNet, resulting in higher network density and average clustering coefficient (which characterizes the overall tendency of nodes to form clusters). Although the network density is similar for both correlation-based methods, the network centralization is much greater for WGCNA than for the two other methods, giving a greater importance to hubs in the network. This topological property is apparent in the networks shown in Fig 4. Network heterogeneity represents the tendency of a network to contain hubs and is similar for all three methods. Finally, the characteristic path length, which represents the average over the shortest paths between all pairs of nodes and offers a measure of a network’s overall navigability, is lower for WGCNA than for the two other methods.

**Table 1:** Network topological properties of regulatory networks inferred using RulNet, WGCNA and Pearson methods.

Topological parameters	RulNet	WGCNA	Pearson
Scale-free *r* ^2^	0.82	0.99	0.81
Absolute scaling exponent γ	1.21	1.41	1.08
no. of nodes	397	238	375
no. of edges	1,562	1,564	2,502
Avg. no. of neighbours	6.36	13.14	13.34
Connected components	14	10	10
Network density	0.02	0.05	0.04
Avg. clustering coefficient	0.27	0.71	0.53
Network centralization	0.13	0.29	0.16
Network heterogeneity	1.17	1.14	1.10
Characteristic path length	3.52	2.71	3.71

**Fig 5:**
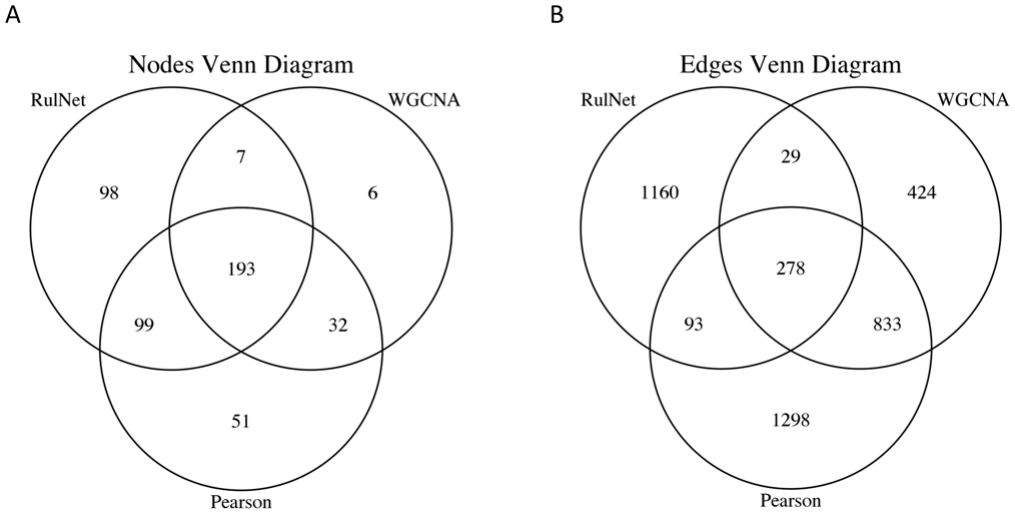
Node and edges comparison between three inference methods. Venn diagram showing node (A) and edge (B) homology between the RulNet platform, WGCNA and Pearson network inference methods.

Out of the total number of 486 nodes and 4,115 edges found with the three network inference methods, 40% of the nodes and only 7% of the edges are conserved in the three methods (Figs 4D and 5). Only 6 nodes are specific to WGCNA while 51 and 98 are specific to Pearson and RulNet, respectively. The conservation of edges between WGCNA and Pearson increases almost linearly with the connectivity of the nodes, from 13% for nodes with a connectivity degree of 1 to 5, to 66% for nodes with a connectivity degree of 81 to 85 (data not shown). The conservation of edges between RulNet and WGCNA and between RulNet and Pearson is independent of the connectivity of the nodes and is close to 10%. The greater conservation of edges between WGCNA and Pearson than between RulNet and the two other methods is not surprising as both WGCNA and Pearson are based on correlation coefficients. These results illustrate the complementarity of RN inference methods reported in previous studies [54].

### Evaluation of RulNet using the DREAM5 network inference challenge datasets

The DREAM5 challenge was a community competition to evaluate network inference methods that predict genome-scale transcriptional regulatory networks from gene-expression microarray datasets. Three standard datasets were used for the comparison taken from a prokaryotic model organism (*E*. *coli*), a eukaryotic model organism (*S*. *cerevisiae*) and an *in silico* generated network [19]. Each of these three datasets comprises a wide range of experimental conditions: genetic perturbations (e.g. gene deletions), drug and environmental perturbations, some microarrays are part of time-series, others are not. Twenty nine teams participated to the DREAM5 challenge and thirty five methods were compared. For each method, participants were asked to provide a ranked list of 100 000 interactions per dataset which was used to assess the performance of the methods.

Two standard quality metrics from machine learning were calculated, the area under the precision-recall (AUPR) and receiver operating characteristic (AUROC) curves [54]. AUPR is a single measure that summarizes the tradeoff between the completeness (recall) and fidelity (precision) of the inferred network, while AUROC is a single measure that summarizes the tradeoff between the rate of true and false positive predicted edges in a gold standard network. Therefore these two metrics are complementary and provide a comprehensive characterization of the predicted networks. To summarize the performance of each individual method across the three networks overall scores were derived from the AUPR and the AUROC scores by calculating the geometric mean of the network specific scores [19, 54]. Finally, an overall score was obtained as the mean of the overall AUPR and AUROC scores. These metrics were calculated using only the interactions for which experimentally supported interactions exists (gold standard interactions). Network predictions from individual teams were integrated to form community networks by rescoring interactions according to their average rank across all methods [19].

We benchmarked RulNet using the three datasets and methodology used in the DREAM5 challenge and compared our results with those from the 35 methods that participated in this challenge [19]. Two queries called QD1 and QD2 were written to take into account the diversity of the experimental conditions (S3 File). QD1 allows the discovery of rules between highly and weakly expressed genes, while QD2 allows the discovery of rules between knockout transcription factors and their putative target genes. QD2 is more reliable since it is based on TF deletion experiments, however it does not concern all transcription factors but only those for which such experiments are available.

For each of these two queries, interactions were ranked by descending leverage and confidence. The best rank obtained with QD1 and QD2 were kept for each interaction, which defines a third method named QD1+2. This new method makes the best out of the two queries QD1 and QD2 that respond to two different questions and will expectedly give better results than each query used alone. The ranked interactions for each dataset and each method are given in Supplementary S1 Dataset to S9 Dataset.

**Fig 6:**
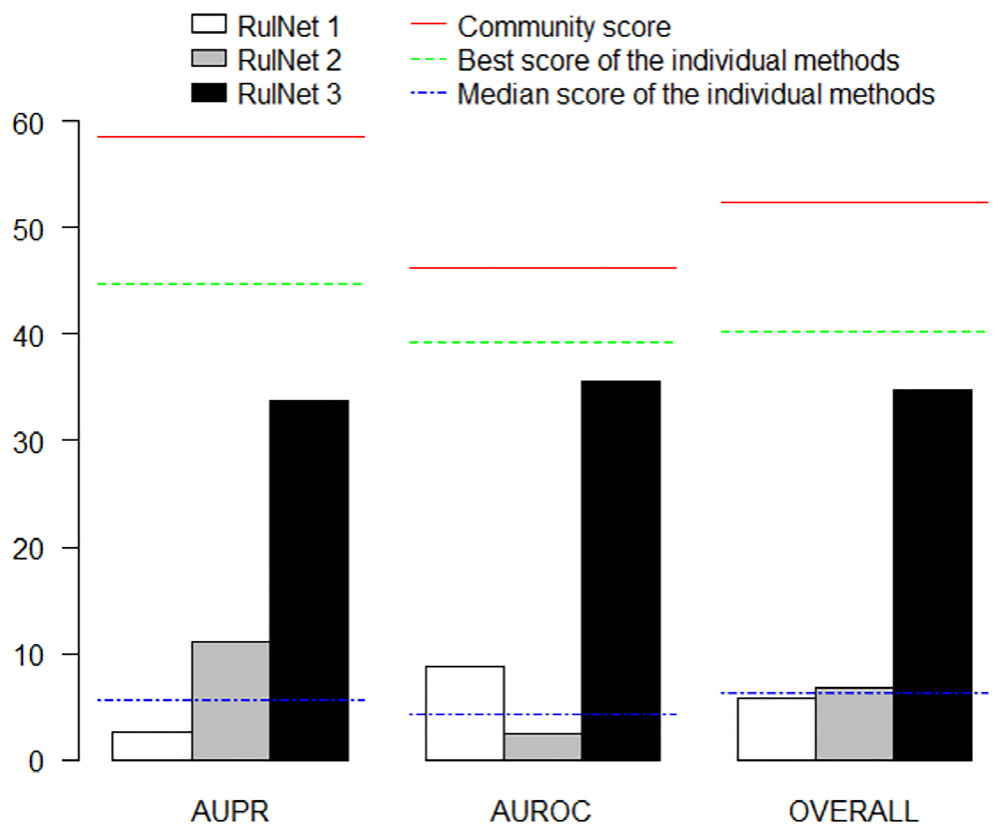
Evaluation of RulNet performance using the DREAM5 network inference challenge datasets. The overall (geometric mean across the three inferred networks) area under the precision-recall (AUPR) and receiver operating characteristic (AUROC) and overall score (mean of the overall AUPR and AUROC scores) obtained using RulNet with the QD1, QD2 and QD1+2 queries (S3 File) were compared to the 35 methods evaluated in the DREAM5 challenge. The horizontal continuous red lines indicate the scores of the integrated community predictions from DREAM5, the vertical dashed green lines indicate the scores of the best individual model from DREAM5 based on the overall score, and the horizontal dash-dot blue lines indicate the median score for 35 methods evaluated in the DREAM5 challenge.

Fig 6 shows the values of the AUPR, AUROC and overall scores over the three networks for the three QD queries. Results for the three individual networks and each individual method of DREAM5 and RulNet are given in S2 Fig QD1 gives higher AUROC scores than QD2, while QD2 gives higher AUPR scores than QD1. This result is related to the biological meaning of the queries and to the fact that the AUROC reflects the quality of the results obtained throughout the full list of interactions while the AUPR is strongly affected by the performance at the top of the prediction list (i.e. by the most reliable interactions), giving a particular importance to the ranking criteria. Foreseeably, the scores obtained with QD1+2 are much higher than those obtained with either QD1 or QD2. The overall AUPR obtained with QD1+2 is close to that of the best individual method evaluated in the context of DREAM5 (Fig 6) and is second to only one of the 35 individual methods (S2 Fig). More importantly, while some methods provide uneven results depending on the dataset, RulNet performs well for each one of them (S2 Fig).

### Identification of transcription factors involved in the transcriptional reprogramming occurring during grain development and in response to N and S supply

The second objective of this study was to demonstrate how RulNet can be used as a biology-driven clustering method using the identification of transcription factors as an example. This demonstration exploits two features of RulNet. The first is a unique feature that enables central attributes to be defined for the rule discovery algorithm. When attributes are defined as central, only rules involving at least one of these attributes are discovered. Central attributes can be of any type. The second feature of RulNet that is exploited in this biology-driven example is the combined use of quantitative and qualitative data.

We demonstrate how RulNet can be used to find TFs involved in the transcriptomic shifts observed between the three major phases of wheat grain development and how they are involved in the adaptive response of wheat grain to N and S deficiency. A particular interest is in how TFs are potentially involved in the regulation of GSP synthesis in wheat. Two queries called QNS1 and QNS2 were written (S2 File) with Support and Confidence thresholds set at 0.15 and 0.90, respectively.

To illustrate this feature of RulNet, N and S deficiency (qualitative data) and the three phases of grain development (qualitative data) were defined as central attributes to discover TFs, GSPs and metabolites (quantitative data) associated with these attributes. Each sample was associated with a phase of development and a level of N and S supply (low or high). Phase 1 of grain development was assigned to samples taken at 10 and 14 days after flowering, phase 2 to samples taken at 18, 24, and 27 days after flowering, and phase 3 to samples taken at 30 and 34 days after flowering. The expression of all 2,891 TFs transcripts spotted on the microarray was used, as well as the quantity per grain of GSPs and metabolites.

**Fig 7:**
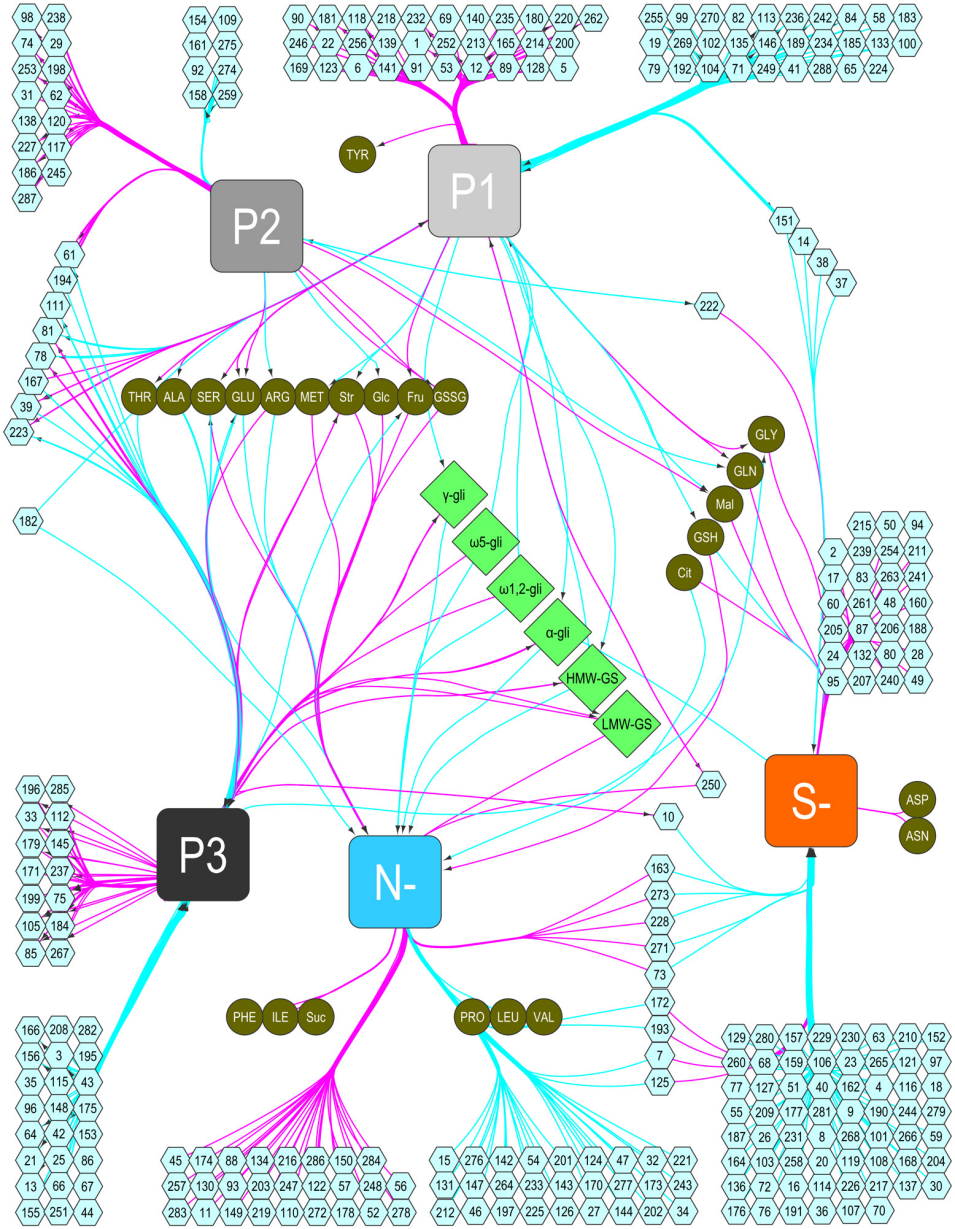
Nitrogen and Sulfur influenced regulatory network in wheat. Directed network inferred using the RulNet platform and illustrating the use of central attributes. Linkages of transcription factors expression (hexagons), and the quantity per grain of storage proteins (squares) and metabolites (circles) with the phases of grain development and nitrogen and sulfur deficiencies defined as central attributes. The network was exported and enhanced in Cytoscape. Nodes were moved and edges were bundled and reorganized for better readability. Pink and light blue edges indicate rules discovered with the QNS1 and QNS2 queries, respectively. The storage proteins ω1,2-, ω5-, γ- and α/β-gliadins (gli) and low (LMW-GS) and high (HMW-GS) molecular weight glutenin subunits were expressed in mg N per grain and the metabolites in μmol per grain. For metabolites and transcription factors, the correspondence between the node id and actual entity names is given in S1 and S2 Tables.

This network can be considered as a biology-driven clustering visualization rather than as a standard interaction network (Fig 7). The edges between attributes imply a functional link between the central attributes and the other attributes rather than a direct interaction. Overall the network highlights TFs transcripts, metabolites and proteins whose relative abundance is linked to the nutrition (N and/or S) or to a particular phase of the grain development or a combination of both. It is also possible to infer interaction between non-central attributes in the same network.

All five GSPs show expected linkages with the early (Phase 1) and late (Phase 3) phases of grain development, being found in low abundance in the early phase and in high abundance during the late phase (Fig 7). Overall, the clustering network obtained through this approach reveals some well-known effects of N and S supply on GSP synthesis [25]. N deficiency implies a low abundance of the four classes of gliadin and HMW-GS while it implies a high abundance of LMW-GS. A S deficiency implies a low abundance of the S-rich α-gliadins.

Regarding the metabolites, many respond to N deficiency but only a few are regulated by S deficiency. For instance, S deficiency implies a high abundance of asparagine (ASN) and aspartate (ASP). It was previously reported that ASN could be increased up to 30-fold in conditions of S deficiency [55–57]. Reduced glutathione (GSH) is also found in low abundance in conditions of S deficiency, while citrate (Cit) and malate (Mal) are found up-regulated, in good agreement with previous reports [58–60].

Besides well know responses of GSPs or metabolites to N and S deficiency, this network reveals putative candidate TFs involved in the molecular response of wheat grains to N and S deficiency. In particular it identifies TFs whose expression is independent of grain development but are down- or up-regulated under conditions of N and/or S deficiency. Sixty-two and 71 TFs are associated with N and S deficiency, respectively, but not with grain development. Among these TFs, nine are associated with both N and S deficiency. Interestingly these TFs show opposite response to N and S, suggesting that they orchestrate the response to the N-to-S balance. For instance, R2R3-MYB_191 is orthologous to *Arabidopsis thaliana* AtMYB7, a TF expressed in seed and involved in hormone mediated signaling pathways (ethylene, abcisic acid, jasmonic acid, salicylic acid) and overall in the response to abiotic stress [61]. C2H2_132 (*A*. *thaliana* orthologue: STOP2) is notably involved in nitrate transport and in the response to intracellular nitrate concentration [61]. WRKY_181 (*A*. *thaliana* orthologue: WRKY30) is involved in the response to ozone and salicylic acid [62] while WRKY_195 (*A*. *thaliana* orthologue: WRKY40) is involved in many pathways including response to hormones, abiotic stress and pathogen defense [61, 63]. All these observations make these TFs good candidates in the context of the study of N/S response in wheat grain. More complex queries could allow us to further elucidate complex regulatory circuitry involved in the adaptation of grain to N/S supply.

## Discussion

### Scale-free topology

Most functional features of a cell are driven by groups of molecules which are not isolated but rather linked to each other or even functionally overlapping. This characteristic is reflected by a scale-free topology of the interaction networks at different levels of cell functional organization. If biological molecules were isolated independent entities the result would be random networks. It is therefore expected that a reliable network inference method would identify this scale-free topology when inferring biological networks. The main feature of scale-free networks is the presence of nodes of widely different connectivity, including weakly connected nodes representing molecules with restricted field of action and hubs (highly connected nodes) representing important regulators. Here we showed that RulNet infers scale-free networks.

### TFs interdependency

We have demonstrated the effectiveness of the RulNet RN inference and visualization platform using—omics data including TF expression. It is well known that TFs are interdependent, i.e. they often work in combination with other TFs. RulNet allows allow discovering rules involving multiple attributes in the left hand side (n-1 MP-rules). Although this feature was not illustrated in the present work it could be used to study combinatorial interactions.

### Programming knowledge

RN inference is an effective tool to predict and analyze RNs using high throughput data. Powerful algorithms and tools have been made available to infer RNs. Unlike many similar tools, programming skills are not required by RulNet users to perform complete network inference, analysis and visualization. Writing simple queries is made easy using the inbuilt query builder, though prior knowledge of the SQL query language would make it easier for users to understand the RQL language for more advanced queries that would exploit all the features in RulNet.

### Dimensionality issue

An important consideration when analyzing data from genome-scale technologies is that technical and experimental limitations do not generally allow the number of observations commensurate with the number of variables (gene transcripts in gene expression studies). In statistical terms-omics studies are usually underpowered. In the general case, the robustness of the results obtained should increase proportionally to the number of observations. The complexity of the genomic-scale approach, however, together with the relatively restricted number of independent data points compared to the size of the search space can limit the robustness of the results. RN inference using—omics data, however, allows researchers to model complex systems of regulation even though they do not reveal the full complexity of the system but only the observable components. Moreover, similar to most approaches to RN inference, users must be cognizant that the interaction inferred between two variables indicates only that their relative quantity are somehow linked; it does not necessarily imply a physical or functional relationship.

### Taking advantage of the complementarity

The main advantage of RulNet stems from its adaptability to different biological problems. This is achieved through its capability of performing different queries in the same software framework. In previous comparative analyses (e.g. DREAM5) it has been shown that most approaches used to infer RNs from the same datasets show relatively distinct results [19]. The differences observed between the inferred RNs does not necessarily demonstrate any poor performance from a particular approach but rather highlights their complementarity. The RulNet approach supported the development of multiple queries to discover rules defining RNs in a unifying framework. These rules were used to evaluate RulNet using the DREAM5 datasets. We showed that the evaluation of the different DREAM5 tests could be improved by exploring different queries. A combined query QD1+2 performed better than that of either QD1 or QD2. This is consistent with the main conclusion of the DREAM consortium, that integrated community networks tend to get closer to a representative network and that this is more likely to be biologically accurate. By supporting the development of combination queries, RulNet supports the inference of integrated networks composed using results from different sub-queries. Based on the quality metrics of the DREAM consortium, we observe that different queries produce different results. This highlights the importance of query design and evaluation prior to the inference process.

The web-oriented platform implemented in RulNet offers multiple features to visualize and edit a global RN by gathering the interactions inferred using different queries. A comparative analysis of RulNet inference method with two other commonly used inference methods, namely correlation network inference and WGCNA, showed that RulNet inference methods produced network topologies that were broadly consistent with other methods. Each method, however, had its own particular behavior reflecting differences in algorithms used. The general conclusion is that when considering the analysis of networks from eukaryotic organisms, it is essential to consider more than one type of interaction. The features in RulNet make this more easy than most of the other available tools. However, it remains the case that no single approach will reveal the absolute and complete truth of complex RNs; only exploiting the complementarity between approaches can narrow the gap between the hypothetical network and the actual complex network of interactions [19].

## Supporting Information

S1 Dataset Ranked interactions for the *in silico* dataset and the QD1 method. (TXT)

S2 Dataset Ranked interactions for the *E*. *coli* dataset and the QD1 method. (TXT)

S3 Dataset Ranked interactions for the *S*. *cerevisiae* dataset and the QD1 method. (TXT)

S4 Dataset Ranked interactions for the *in silico* dataset and the QD2 method. (TXT)

S5 Dataset Ranked interactions for the *E*. *coli* dataset and the QD2 method. (TXT)

S6 Dataset Ranked interactions for the *S*. *cerevisiae* dataset and the QD2 method. (TXT)

S7 Dataset Ranked interactions for the *in silico* dataset and the QD3 method. (TXT)

S8 Dataset Ranked interactions for the *E*. *coli* dataset and the QD3 method. (TXT)

S9 Dataset Ranked interactions for the *S*. *cerevisiae* dataset and the QD3 method. (TXT)

S1 Fig Power law analysis of regulatory networks. (TIF)

S2 Fig Comparison of the performance of RulNet for the DREAM5 networks with that of the 35 network inference methods of DREAM5 challenge. (TIF)

S1 File Grammar specifications of the RQL language. (PDF)

S2 File Semantics used to infer regulatory networks from wheat —omics data. (PDF)

S3 File Semantics used to infer regulatory networks from the DREAM5 datasets. (PDF)

S1 Table Abbreviations of metabolites used in Fig 6. (PDF)

S2 Table Correspondence of transcription factors identifiers and names used in Fig 6. (PDF)
