# Supplementary figures and images for: RulNet: A Web-Oriented Platform for Regulatory Network Inference, Application to Wheat –Omics Data
Source: PLoS One. 2015 May 19;10(5):e0127127. doi: 10.1371/journal.pone.0127127 (PMC4437996; doi:10.1371/journal.pone.0127127)

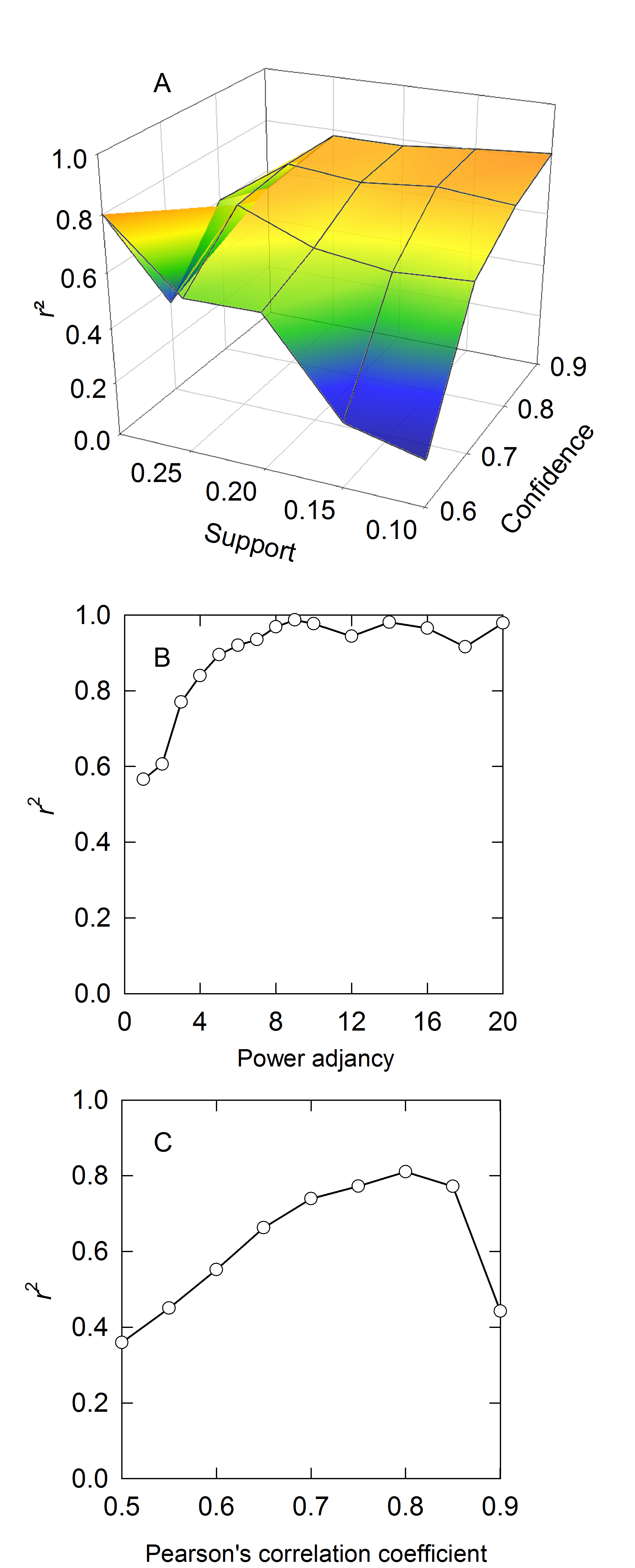

Supplement: S1 Fig — (TIF) [file pone.0127127.s010.tif]

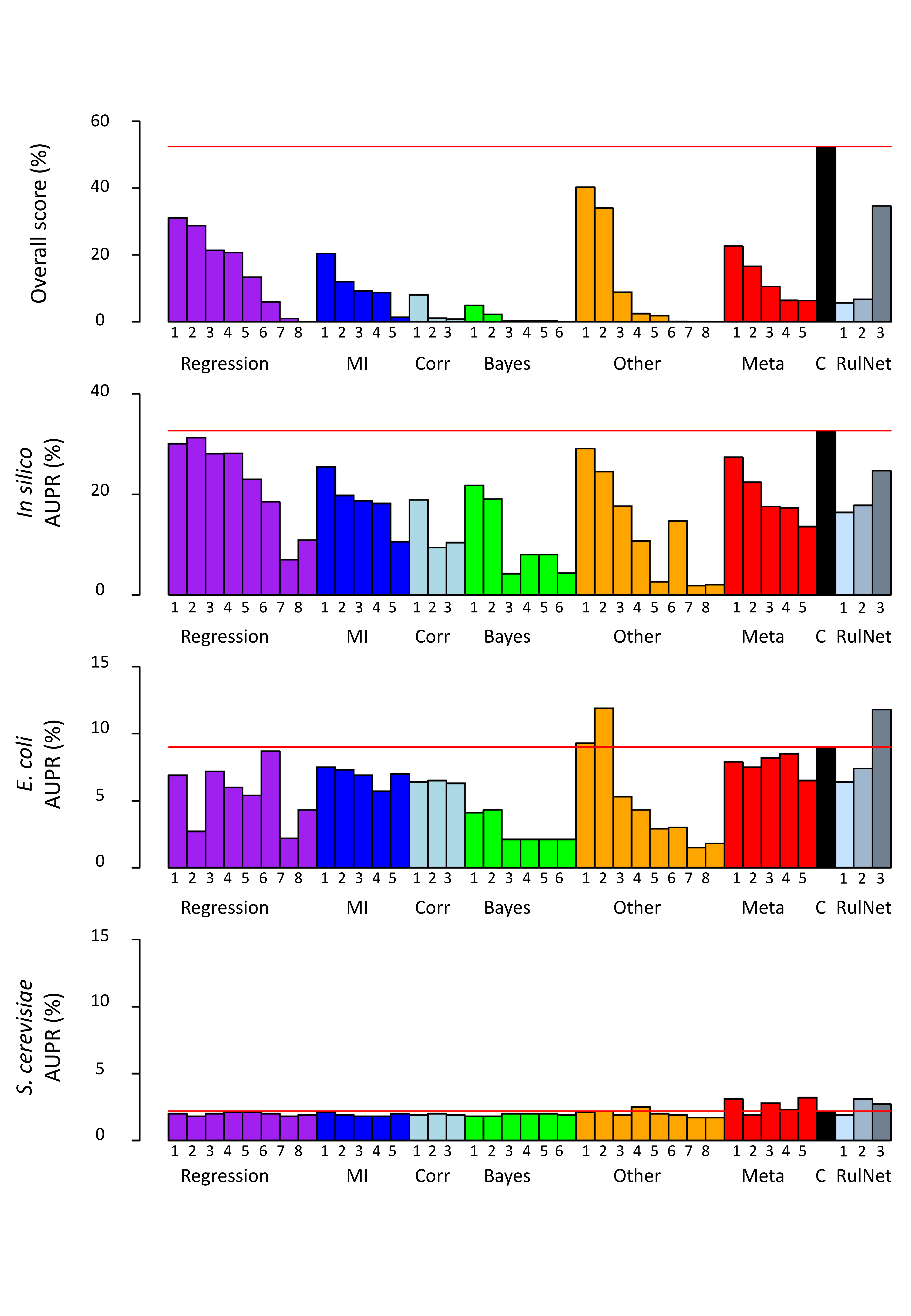

Supplement: S2 Fig — (TIF) [file pone.0127127.s011.tif]
