# Supplementary material for: RulNet: A Web-Oriented Platform for Regulatory Network Inference, Application to Wheat –Omics Data
Source: PLoS One. 2015 May 19;10(5):e0127127. doi: 10.1371/journal.pone.0127127 (PMC4437996; doi:10.1371/journal.pone.0127127)
Supplement: S1 File — (PDF) [file pone.0127127.s012.pdf]

S1 File

Grammar specifications of the RQL language

The grammar specifications of the language to define different semantics can be defined using Backus-Naur Form (BNF) standards (Backus *et al.*, 1960) (Box S1). A BNF consists of a set of derivation rules of the form <*symbol*> ::= _expression_, where _expression_ consists of one or more sequences of symbols, separated by a vertical bar (|) indicating a choice. A symbol is terminal if it does not appear on the left hand side of any derivation rule. The non-terminal symbol <*query*> shows the global form of a query, from which we can see the SQL inspiration.

Box S1. Backus-Naur Form grammar of the extended form of the RQL language

<query> ::=
"FINDRULES "
"SCOPE " <psi>
"HAVING " <pred_list>";"
<psi> ::=
<tupvar_list> " [WHERE " <SQL_condition>]
<tupvar_list> ::=
<tupvar_def> | <tupvar_def> " , " <tupvar_list>
<tupvar_def> ::=
<tupvar> " IN " <data>
<data> ::=
<table_name> | "("<SQL_query>")"
<pred_list> :=
<predicate> | <predicate> " AND " <pred_list>
<predicate> := [<label>" : "] <delta> " OVER " <att_list> | ALL | ALL MINUS <att_list>";"
<delta> ::= <delta_atomic> |
"(" <delta> ")" |
" NOT " <delta> |
<delta> <logical_connector> <delta>
<delta_atomic> ::=
<tupvar> ".ATT " <comparison_op> <tupvar> ".ATT" |
<tupvar> ".ATT " <comparison_op> <constant>
<logical_connector> ::= " OR " | " AND "
<comparison_op> :: "=" | "<" | ">" | "<=" | ">=" | "!="

<*SQL_query*> represents a classical (SELECT FROM WHERE) query and <*SQL_condition*> is the clause used for instance in the "WHERE" statement of a SQL query. RQL also provides most of the functions provided by SQL, such as SQRT, ABS, ROWID, SUBSTR or LTRIM.

The "OVER" clause specifies the attributes on which the predicates have to be discovered. These attributes have to be present in the datasets otherwise the query cannot be semantically correct.

A predicate is defined as a label (<label>), a property (<delta>) and an attribute list (<att_list>) on which the predicate is tested. The clause <label> is optional but it is necessary if multiple predicates are defined for an attribute. The keyword "ATT" will then be replaced by each attribute of the <att_list> clause and tested to check if the rule is satisfied.
