# Supplementary material for: RulNet: A Web-Oriented Platform for Regulatory Network Inference, Application to Wheat –Omics Data
Source: PLoS One. 2015 May 19;10(5):e0127127. doi: 10.1371/journal.pone.0127127 (PMC4437996; doi:10.1371/journal.pone.0127127)
Supplement: S2 File — (PDF) [file pone.0127127.s013.pdf]

S2 File

Semantics used to infer regulatory networks from wheat –omics data

QNS1: Discover rules between highly abundant molecules and N/S supply or development phase.

```
FINDRULES
SCOPE t1 IN Omic_data
HAVING high : t1.ATT > 0.5 OVER ALL MINUS sample, Nitrogen, Sulfur, Phase_1,
Phase_2, Phase_3
AND NoNitrogen : t1.ATT = 0 OVER Nitrogen
AND Nitrogen : t1.ATT = 15 OVER Nitrogen
AND NoSulfur : t1.ATT = 0.2 OVER Sulfur
AND Sulfur: t1.ATT = 2 OVER Sulfur
AND Cellularization : t1.ATT = 1 OVER Phase_1
AND Storage : t1.ATT = 1 OVER Phase_2
AND Senescence : t1.ATT = 1 OVER Phase_3;
```

QNS2: Discover rules between not abundant molecules and N/S supply or development phase.

```
FINDRULES
SCOPE t1 IN Omic_data
HAVING high : t1.ATT < -0.5 OVER ALL MINUS sample, Nitrogen, Sulfur, Phase_1,
Phase_2, Phase_3
AND NoNitrogen : t1.ATT = 0 OVER Nitrogen
AND Nitrogen : t1.ATT=15 OVER Nitrogen
AND NoSulfur : t1.ATT = 0.2 OVER Sulfur
AND Sulfur: t1.ATT = 2 OVER Sulfur
AND Cellularization : t1.ATT = 1 OVER Phase_1
AND Storage : t1.ATT = 1 OVER Phase_2
AND Senescence : t1.ATT = 1 OVER Phase_3;
```
