# Supplementary material for: RulNet: A Web-Oriented Platform for Regulatory Network Inference, Application to Wheat –Omics Data
Source: PLoS One. 2015 May 19;10(5):e0127127. doi: 10.1371/journal.pone.0127127 (PMC4437996; doi:10.1371/journal.pone.0127127)
Supplement: S3 File — (PDF) [file pone.0127127.s014.pdf]

S3 File.

Semantics used to infer regulatory networks from the DREAM5 datasets

QD1: Discover rules between highly and weakly expressed genes.

```
FINDRULES
SCOPE t1 IN netx
HAVING high : t1.ATT > 0.5 OVER ALL
AND low : t1.ATT < -0.5 OVER ALL
```

QD2: Discover rules between knockout transcription factors and their putative target genes.

```
FINDRULES
SCOPE t1 IN netx, t2 IN netx
WHERE t1.Experiment = t2.Experiment AND t1.Perturbations = t2.Perturbations AND
t1.PerturbationLevels = t2.PerturbationLevels AND t1.Treatment = t2.Treatment AND
t1.OverexpressedGenes = t2.OverexpressedGenes AND t1.Time = t2.Time AND
t1.DeletedGenes != t2.DeletedGenes AND t1.DeletedGenes = 'NA'
HAVING higher : t2.ATT-t1.ATT > 0.5 OVER ALL
AND lower : t1.ATT-t2.ATT > 0.5 OVER ALL
AND G1 : t2.ATT='G1' OVER DeletedGenes
AND …
AND G195 : t2.ATT='G195' OVER DeletedGenes
```
