# Supplementary material for: RulNet: A Web-Oriented Platform for Regulatory Network Inference, Application to Wheat –Omics Data
Source: PLoS One. 2015 May 19;10(5):e0127127. doi: 10.1371/journal.pone.0127127 (PMC4437996; doi:10.1371/journal.pone.0127127)
Supplement: S1 Table — (PDF) [file pone.0127127.s015.pdf]

S1 Table. Abbreviations of metabolites used in Figure 6.

**Metabolite**	**Abbreviation**
Alanine	ALA
Arginine	ARG
Asparagine	ASN
Aspartic acid	ASP
Glutamine	GLN
Glutamic acid	GLU
Glycine	GLY
Reduced Glutathione	GSH
Glutathione disulfide	GSSG
Isoleucine	ILE
Leucine	LEU
Methionine	MET
Phenylalanine	PHE
Proline	PRO
Serine	SER
Threonine	THR
Tyrosine	TYR
Valine	VAL
Citrate	Cit
Fructose	Fru
Glucose	Glc
Malate	Mal
Starch	Str
Sucrose	Suc
