# Supplementary material for: RulNet: A Web-Oriented Platform for Regulatory Network Inference, Application to Wheat –Omics Data
Source: PLoS One. 2015 May 19;10(5):e0127127. doi: 10.1371/journal.pone.0127127 (PMC4437996; doi:10.1371/journal.pone.0127127)
Supplement: S2 Table — (PDF) [file pone.0127127.s016.pdf]

S2 Table. Correspondence of transcription factors identifiers and names used in Figure 6. The names are those used in the wheat transcription factor database wDBTF (http://wwwappli.nantes.inra.fr:8180/wDBFT/).

Id	Name	Id	Name	Id	Names
1	Alfin_8	40	bHLH_90	79	C3H_106
2	AP2_16	41	bZIP_117	80	C3H_32
3	ARF_48	42	bZIP_131	81	C3H_4
4	ARF_49	43	bZIP_133	82	C3H_49
5	ARF_77	44	bZIP_134	83	C3H_59
6	ARF_80	45	bZIP_139	84	C3H_93
7	ARID_3	46	bZIP_147	85	CCAAT-HAP2_7
8	AS2_16	47	bZIP_153	86	CCAAT-HAP3_1
9	AS2_20	48	bZIP_157	87	CCAAT-HAP3_2
10	AS2_21	49	bZIP_162	88	CCAAT-HAP5_18
11	AS2_5	50	bZIP_163	89	CPP_16
12	ASH1_3	51	bZIP_35	90	CPP_3
13	AUX/IAA_1	52	bZIP_53	91	E2F-DP_11
14	AUX/IAA_14	53	bZIP_80	92	E2F-DP_4
15	AUX/IAA_57	54	bZIP_91	93	EIL_11
16	AUX/IAA_75	55	bZIP_94	94	EIL_16
17	BBR/BPC_3	56	C2C2-CO-like_64	95	EIL_19
18	BES1_10	57	C2C2-CO-like_71	96	EREBP_102
19	BES1_9	58	C2C2-CO-like_8	97	EREBP_106
20	BHL_13	59	C2C2-CO-like_89	98	EREBP_108
21	BHL_16	60	C2C2-DOF_35	99	EREBP_149
22	BHL_20	61	C2C2-DOF_9	100	EREBP_153
23	BHL_6	62	C2C2-GATA_10	101	EREBP_157
24	bHLH_119	63	C2C2-GATA_13	102	EREBP_159
25	bHLH_120	64	C2C2-GATA_17	103	EREBP_16
26	bHLH_153	65	C2C2-GATA_20	104	EREBP_161
27	bHLH_161	66	C2C2-GATA_21	105	EREBP_162
28	bHLH_165	67	C2C2-YABBY_10	106	EREBP_163
29	bHLH_171	68	C2C2-YABBY_4	107	EREBP_176
30	bHLH_173	69	C2H2_102	108	EREBP_191
31	bHLH_184	70	C2H2_103	109	EREBP_197
32	bHLH_222	71	C2H2_118	110	EREBP_2
33	bHLH_23	72	C2H2_121	111	EREBP_206
34	bHLH_265	73	C2H2_132	112	EREBP_213
35	bHLH_278	74	C2H2_138	113	EREBP_225
36	bHLH_283	75	C2H2_170	114	EREBP_232
37	bHLH_302	76	C2H2_74	115	EREBP_246
38	bHLH_35	77	C2H2_80	116	EREBP_267
39	bHLH_69	78	C3H_10	117	EREBP_39

S2 Table. Continued

118	EREBP_42	157	M-type_2	196	NAC_32
119	EREBP_56	158	MBF1_4	197	NAC_34
120	EREBP_58	159	MBF1_43	198	NAC_90
121	EREBP_68	160	MBF1_9	199	NAC_95
122	EREBP_86	161	MIKC-type_13	200	Nin-like_2
123	GARP-ARR-B_19	162	MIKC-type_32	201	PAT1_2
124	GARP-G2-like_22	163	MIKC-type_4	202	PAT1_4
125	GARP-G2-like_23	164	MIKC-type_63	203	PAT1_9
126	GARP-G2-like_4	165	MIKC-type_7	204	PHD-ZNFG_1
127	GARP-G2-like_79	166	MIKC-type_87	205	PHD-ZNFG_146
128	GARP-G2-like_85	167	MIKC-type_96	206	PHD-ZNFG_149
129	GARP-G2-like_89	168	MYB-related_11	207	PHD-ZNFG_186
130	GeBP_16	169	MYB-related_114	208	PHD-ZNFG_201
131	GeBP_7	170	MYB-related_125	209	PHD-ZNFG_211
132	GeBP_8	171	MYB-related_126	210	PHD-ZNFG_244
133	HAM_11	172	MYB-related_162	211	PHD-ZNFG_247
134	HAM_3	173	MYB-related_27	212	PHD-ZNFG_248
135	HD-ZIP_14	174	MYB-related_29	213	PHD-ZNFG_261
136	HD-ZIP_17	175	MYB-related_36	214	PHD-ZNFG_262
137	HD-ZIP_32	176	MYB-related_7	215	PHD-ZNFG_287
138	HD-ZIP_6	177	MYB-related_98	216	PHD-ZNFG_289
139	HD-ZIP_88	178	MYB-related_99	217	PHD-ZNFG_60
140	HMG_46	179	NAC_109	218	PHD-ZNFG_61
141	HMG_47	180	NAC_118	219	PHD-ZNFG_71
142	HMG_50	181	NAC_119	220	PHD-ZNFG_73
143	HMG_77	182	NAC_12	221	PHD-ZNFG_77
144	HMG_81	183	NAC_125	222	PHD-ZNFG_83
145	HSF_12	184	NAC_126	223	PLATZ_1
146	HSF_48	185	NAC_13	224	R2R3-MYB_105
147	HSF_54	186	NAC_14	225	R2R3-MYB_132
148	HSF_58	187	NAC_159	226	R2R3-MYB_166
149	JUMONJI_13	188	NAC_16	227	R2R3-MYB_181
150	KNOX_15	189	NAC_172	228	R2R3-MYB_191
151	KNOX_17	190	NAC_20	229	R2R3-MYB_193
152	LIM_10	191	NAC_203	230	R2R3-MYB_208
153	LIM_28	192	NAC_222	231	R2R3-MYB_210
154	LIM_36	193	NAC_244	232	R2R3-MYB_219
155	LUG_13	194	NAC_249	233	R2R3-MYB_232
156	LUG_14	195	NAC_250	234	R2R3-MYB_27

S2 Table. Continued

235	R2R3-MYB_31	274	WRKY_199
236	R2R3-MYB_83	275	WRKY_201
237	REM_12	276	WRKY_210
238	REM_18	277	WRKY_215
239	REM_30	278	WRKY_222
240	REM_46	279	WRKY_31
241	REM_54	280	WRKY_46
242	REM_57	281	WRKY_81
243	REM_62	282	WRKY_82
244	REM_65	283	WRKY_87
245	REM_66	284	ZF-HD_5
246	REM_7	285	ZF-HD_9
247	REM_75	286	ZIM_20
248	SBP_22	287	ZIM_4
249	SBP_24	288	ZIM_75
250	SBP_5		
251	SUVAR_24		
252	SUVAR_40		
253	TAZ_13		
254	Trihelix_1		
255	Trihelix_2		
256	Trihelix_24		
257	Trihelix_33		
258	Trihelix_37		
259	TUB_28		
260	TUB_29		
261	TUB_41		
262	TUB_9		
263	Whirly_4		
264	WRKY_113		
265	WRKY_120		
266	WRKY_137		
267	WRKY_150		
268	WRKY_17		
269	WRKY_171		
270	WRKY_173		
271	WRKY_181		
272	WRKY_190		
273	WRKY_195		
